# Faith Spicer OBE (MB, BS, JP): Formerly Director, London Youth Advisory Centre (later the Brandon Centre)

**DOI:** 10.1192/pb.bp.115.051482

**Published:** 2016-02

**Authors:** Peter Wilson

**Figure:**
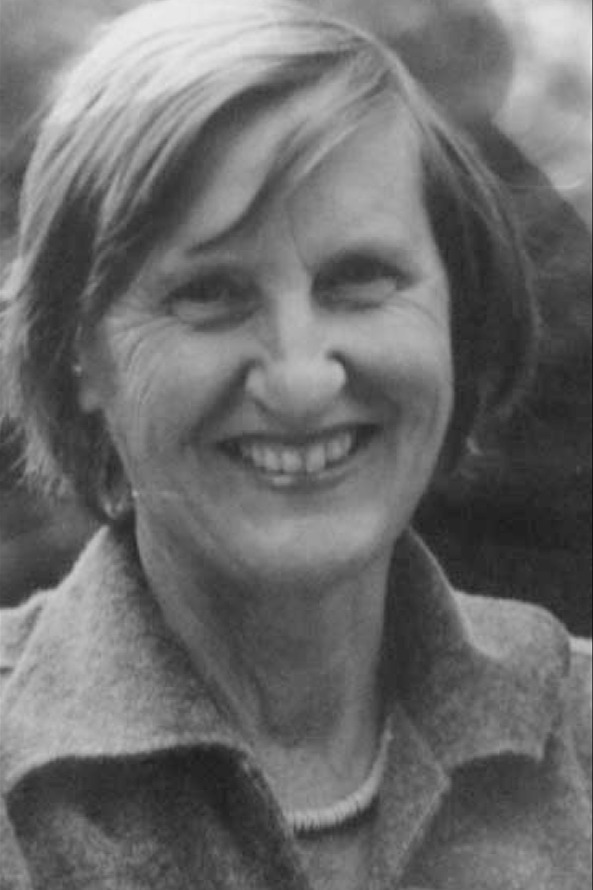


Dr Faith Spicer, who recently died at the age of 95, was a truly resolute pioneer in the fields of family planning, sexual counselling and the psychiatry of adolescence. She was acutely aware of the shame and moral outrage that in her time was associated with pre-marital sexual intercourse and its often appalling consequences, not least dangerous, back-street abortions. At that time, women students who became pregnant were seeking abortions for fear of their being sent down. Illegitimate birth rates were accelerating at an alarming rate.

Something needed to be done to challenge the prevailing prejudices and it was testimony to Faith's courage that she entered so powerfully into the fray. In recognition of the predicament of young women who were struggling with their sexuality in a changing society of the 1950s and early 1960s, she became a vigorous campaigner on their behalf for greater public awareness and improved psychosexual services. Her work was controversial, occurring as it did in what were very unsettling times for many people. Religious and moral pressures to uphold the virtues of virginity before marriage were slowly having to face, and adjust to the emergence of, a spirit of greater sexual freedom, which was soon to be further facilitated by the impact of the new technology of its time, the oral contraceptive pill. Faith was viewed with much suspicion but also greeted, especially by the young, with relief and gratitude.

She first practised contraceptive counselling in the Marie Stopes Clinic, whose mission was to prevent unwanted pregnancies. In 1963 she was appointed Medical Director of the Brook Advisory Centre. After 4 years she resigned from that post because she objected to those in management who wanted to speed up the process of contraceptive advice and increase the number of young people being seen. Faith took a more measured view of the work that was required, arguing that consultations should not be rushed. The issue for her was not so much that of the contraceptive pill itself as of the emotional confusion and turmoil that was often associated with sexual life and development. In 1969 she founded the London Youth Advisory Centre (LYAC), later renamed the Brandon Centre and eventually sited in Kentish Town, London. This established a new model of service for adolescents from the age of 12 up to 25, combining contraceptive advice and counselling/psychotherapy. Dr Gill Hinshelwood, one of Faith's closest colleagues in the LYAC, recalls the birth and growth of the centre at that time as inspiring and indeed revelatory: ‘All the most exciting and dangerous things happen on borders and work at LYAC crossed the boundaries of many medical disciplines, including psychiatry, gynaecology, social work, paediatrics and psychotherapy. It was very challenging.’

As time went on Faith became increasingly successful in combating ignorance and fear among the young. She understood the anxiety and uncertainty that many experienced in making decisions in their sexual lives – so often risking their future either by embarking on experiences they were not ready for or refusing experiences they should have. She gave many talks and lectures on a wide range of subjects concerning young people and wrote two important books, *Sex and the Love Relationship* (1972, Priory Press) and *Adolescence and Stress* (1977, Forbes Publications). These admirably convey her knowledge and commitment to her cause as well as her compassionate understanding of those who, as children, had been deeply affected by deprivation and abuse. ‘These young people’, she wrote, ‘have every right to dislike our society and to try to find something better’.

Dr Spicer was born one of twins, the youngest of nine children of the Rev. Montague Gifford James and his wife Violet. Her mother encouraged her daughters to be independent and creative women and Faith did not fail to live up to her expectations. She completed her medical training in 1944 at University College London and became a psychiatrist specialising in work with adolescents and psychosexual medicine. She married a fellow doctor, Clive Spicer, when she was 22 and raised a family of three children. Her marriage ended in divorce in 1986 and she later married Tony Estill who died in 2002.

After her retirement at the age of 65, she became a consultant at the Anna Freud Centre and to the Cotswold Community, a residential therapeutic community for disturbed adolescents. She also served as chair of a Juvenile Magistrates Court and as a member of the British Board of Film Classification.

People who knew Faith enjoyed her warmth and generosity as well as her sharp intelligence and lively personality. They knew too that at times she could be quite forthright and unequivocal in fighting for what she thought right. The LYAC could not have been born or grown without these qualities in her leadership. There have been many who have been greatly influenced and helped by her. It is particularly appropriate that, not far from where Faith carried out so much of her work, one of her granddaughters, Nell Nicholson, runs Gloucester House, the Tavistock Children's Day Unit carrying out therapeutic care of vulnerable young people.

Dr Faith Spicer died on 22 December 2014. She is survived by her three children, Jane, Mary and David, five grandchildren and four great-grandchildren.

